# Clinical efficacy of Vortioxetine and escitalopram in the treatment of depression

**DOI:** 10.12669/pjms.38.5.5230

**Published:** 2022

**Authors:** Shichuan Shao, Baomin Sun, Huiqing Sun

**Affiliations:** 1 Shichuan Shao Department of Medical Administration, Tai’an City Central Hospital, Taian, Shandong, 271000, China; 2 Baomin Sun Department of Clinical Psychology, Tai’an City Central Hospital, Taian, Shandong, 271000, China; 3 Huiqing Sun Department of Pharmacy, Tai’an City Central Hospital, Taian, Shandong, 271000, China

**Keywords:** Vortioxetine, Escitalopram, Major depressive disorder, Clinical efficacy

## Abstract

**Objectives:**

This study was aimed to investigate the efficacy and safety of vortioxetine hydrobromide in the treatment of major depressive disorder (MDD).

**Methods:**

One hundred and eighty patients with the newly diagnosed depression in our hospital between August 2018 and August 2019 were selected and randomly divided into an observation group and a control group, 90 each group. The control group was treated with escitalopram, and the observation group was treated with voltaxetine. The efficacy and adverse reactions were evaluated by the Hamilton Depression scale-17 (HAMD-17), Sheehan Disability Scale (SDS), Perceived Deficits Questionnaire-Depression (PDQ-D), and treatment emergent symptom scale (TESS) before treatment and at the end of the 8th and 24th week after treatment.

**Results:**

At the end of the 8th and 24th week after treatment, the HAMD-17 scores of the two groups were lower than those before treatment (P<0.05); at the end of the 8th and 24th week after treatment, the PDQ-D and SDS scores of the two groups were lower than those before treatment (P<0.05), and the above scores of the observation group were lower than those of the control group (P<0.05). There was no significant difference in the incidence of adverse reactions between the two groups (P>0.05).

**Conclusion:**

Voltaxetine can improve cognitive function and clinical symptoms of patients with severe depression and has high safety, which is worth clinical attention.

## INTRODUCTION

Depressive disorder is a highly prevalent, chronic and recurrent disorder. It is currently ranked as one of the leading causes of disability worldwide, affecting approximately 350 million people worldwide.^1^ The main core symptoms of depressive disorder are persistent low mood, lack of interest and reduced energy,^2^ which can severely affect the social functioning of people and create a significant disease burden.^3^ In addition, patients with depressive disorders often have widespread and pervasive cognitive impairment.^4^,^5^ According to the American Diagnostic and Statistical Manual of Mental Disorders (DSM-4 & DSM-5), cognitive symptoms are one of the nine criteria for diagnosing depressive episodes.^6^ The persistence of cognitive impairment after improvement of depressive symptoms has been shown to be one of the important reasons for the incomplete recovery of social functioning in patients with depressive disorders.^7^,^8^

Medication is still a major clinical treatment for depressive disorders. In recent years, antidepressants with various mechanisms of action have emerged, for example, escitalopram, which was launched in 2002, has been widely recognized by the industry for its efficacy and safety in the treatment of patients with depressive disorders as a selective serotonin reuptake inhibitor (SSRI). In a double-blind, placebo-controlled clinical trial that included 380 patients with depressive disorders, escitalopram at a dose of 10 mg/d for two weeks was already efficacious compared to placebo.^9^ Another study examined the long-term pharmacological treatment of depressive disorders in a randomized, double-blind trial of 274 patients with depressive disorders over a 36-week period. After eight weeks of treatment, the intervention was continued with escitalopram maintenance, and the patients’ total Montgomery-Asberg Depression Rating Scale (MADRS) scores further reduced at the end of the 36th week, confirming the superior clinical outcome of long-term treatment.^10^

With the continuous research and development of new antidepressants in recent years, a variety of new antidepressants have been constantly used in psychiatric clinic, and votioxetine is one of them. Votioxetine was listed in the United States in 2013 and China in November 2017. Votioxetine is a new multi-mode antidepressant, which exerts antidepressant effect by acting on six pharmacological targets ((antagonizing 5-HT3, antagonizing 5-HT7, antagonizing 5-HT1D, partially activating 5-HT1B, activating 5-HT1A, and inhibiting serotonin transporter (SERT)) through two different action modes (inhibiting the reuptake of 5-HT transporter SERT, and regulating 5-HT receptor). As votioxetine has the ability to regulate norepinephrine, dopaminergic, cholinergic, histaminergic, glutamine, and aminobutyric acid systems, it may play its role by regulating the neural circuit.^11^ Studies overseas have confirmed that votioxetine has ideal efficacy and safety in the treatment of depressive disorder and its improvement in cognitive function and social function of patients is worth affirmation.^12^,^13^ A study included 156 elderly patients over 65 years of age with depressive disorders and treated them with vortioxetine for eight weeks.^14^ The efficacy of vortioxetine was significantly effective than placebo, and it was concluded that vortioxetine significantly improved cognitive function in elderly patients by two neuropsychological tests, the Digit Symbol Substitution Test (DSST) and the Rey Auditory Verbal Learning Test (RAVLT).

Since vortioxetine was launched in China in 2017, only a few studies have been conducted in China to investigate the efficacy and safety of the drug.^15^ However, the dimensions of efficacy evaluation in these studies were only limited to depressive mood, while the cognitive and social functions of patients were not discussed in detail; moreover, no randomized controlled studies were conducted for escitalopram. Accordingly, this study comprehensively investigated the efficacy and safety of vortioxetine and escitalopram in the treatment of patients with depressive disorders from multiple perspectives. The aim of this study was to expand the evidence-based evidence of these two antidepressants in a more in-depth and comprehensive manner to guide clinical use.

## METHODS

Patients with major depressive disorder (MDD) who were admitted to and treated in our hospital from August 2018 to August 2019 were selected. The study was approved by the medical ethics committee of our hospital, and the informed consent has been signed by the subjects.

### Inclusive criteria:

- Aged 18-55 years;
- Meeting the diagnostic criteria of MDD in ICD-10;
- Scores of 17 items of HAMD ≥ 18. Patients with severe physical, mental illness, and alcohol and drug abuse were excluded. One hundred and eighty patients were enrolled, and all of them completed the whole research process.

All patients were randomly divided into an observation group and a control group according to the random number table method. The observation group included 90 cases, 39 males and 51 females; the average age was (36.79±13.51) years old, the average course of disease was (10.22±9.05) months. The control group included 90 cases, 30 males and 60 females; the average age was (40.76±13.30) years old, and the average course of disease was (15.32±33.18) months. There was no significant difference in the general data between the two groups (P>0.05).

Both groups received the conventional psychiatric treatment and nursing scheme. The observation group was treated with the systemic treatment of voltaxetine tablets. The patient took the medicine once in the morning. The initial dose was 5 mg/d, and then the dose was increased to 10 mg/d according to the specific clinical situation. The control group was treated by systematic treatment of escitalopram tablets, once in the morning, the initial dose was 10 mg/d, and then the dosage was increased to 20 mg/d according to the clinical conditions. In the whole research process.

Other kinds of antidepressants, antipsychotics, mood stabilizers and anti-anxiety drugs were prohibited; physical therapy methods such as modified electroconvulsive therapy (MECT) and transcranial magnetic stimulation were prohibited; psychotherapy methods such as psychoanalysis, behavior therapy and cognitive therapy were prohibited; if the patients had obvious sleep disorders, alprazolam at a dosage of 0.04 mg/d was used; during the study, other drugs other than those specified were prohibited. Both groups were treated for 24 weeks.

### Hamilton Depression scale-17 (HAMD-17):^16^

it is used for evaluating the depressive symptoms of patients, and it is composed of five factors: anxiety/omatization, weight, cognitive impairment, retardation, and sleep disorders; the milder the disease condition is, the lower the score is. *Sheehan Disability Scale (SDS):^17^* the scale was developed by Sheehan in 1983, which can not only evaluate the disability and functional impairment caused by a variety of chronic diseases (including mental disorders) but also evaluate the improvement of social function of patients in clinical efficacy research; it is a self-rating scale, which is divided into three dimensions: work/study, social life, and family life/family responsibility; each dimension can be rated as 0-10 points according to the degree of disease impact, 0 point for completely not affected and 10 points for extremely affected; the total score is the sum of three scores; the lower the score is, the milder the impairment on the social function is; it has favourable internal consistency and good reliability and validity. *Perceived Deficits Questionnaire-Depression (PDQ-D):^18^* it is used for evaluating the severity of cognitive impairment of patients with depression disorders; it is composed of 20 items, 0 ~ 4 points for each item; the lower the score is, the milder the cognitive impairment is. (4) The occurrence of adverse reactions in patients was observed, and the adverse reactions were determined according to the treatment emergent symptom scale (TESS).

The efficacy and safety were evaluated by the HAMD-17, PDQ-D and TESS before treatment and at the end of the 8th and 24th weeks after treatment. The level of social function of the two groups of patients before treatment and at the end of the 8th and 24th weeks after treatment was evaluated by SDS. The assessors were at least attending doctors and have been trained uniformly. The Kappa value in the consistency check was 0.89.

### Statistical Analysis:

All data were processed by SPSS 20.0 software. If the measurement data were normally distributed, the comparison between groups was performed by the independent sample t test, and the data were presented in the form of Mean±SD. The count data was treated by Chi-square test and expressed in the form of [n (%)]. The difference was statistically significant with P<0.05.

## RESULTS

**Fig.1:**
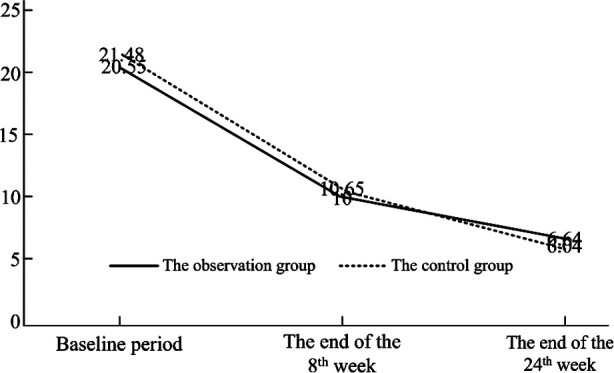
The variation trend of the HAMD-17 score of two groups in different treatment stages.

Before receiving treatment, the total HAMD-17 score did not show a significant difference between the two groups (P>0.05). At the end of the 8th and 24th weeks after treatment, the total HAMD-17 scores in both groups were significantly lower than those before treatment (P<0.05); there was no significant difference in the total HAMD-17 score between the two groups (P>0.05) (Table-I and Fig.1).

**Table I:** Comparison of HAMD-17 score between two groups in different treatment stages (χ¯±S point).

Treatment stage	Observation group (n=90)	Control group (n=90)	t	P
Before treatment	20.56±7.33	21.48±4.87	0.767	0.451
At the end of the 8^th^ week	10.01±5.48^*^	10.65±4.11^*^	0.685	0.492
At the end of the 24^th^ week	6.63±3.44^*^	6.04±2.23^*^	1.059	0.286

Note:

* :P<0.05 compared to before treatment.

**Fig.2:**
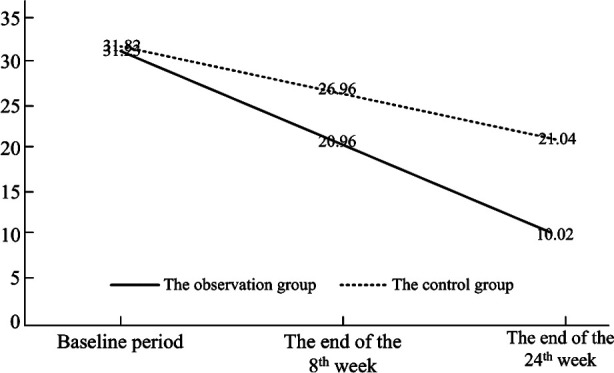
The variation trend of the PDQ-D score of two groups in different treatment stages.

Before treatment, the PDQ-D score did not show a significant difference between the two groups (P>0.05). At the end of the 8th and 24th weeks after treatment, the PDQ-D score in both groups were significantly lower than those before treatment (P<0.05); the PDQ-D score of the observation group was significantly lower than that of the control group (P<0.05) (Table-II and Fig.2).

**Table II:** Comparison of PDQ-D score between two groups in different treatment stages (χ¯±Spoint).

Treatment stage	Observation group (n=90)	Control group (n=90)	t	P
Before treatment	31.24±16.06	31.93±13.18	0.243	0.810
At the end of the 8^th^ week	20.95±13.98^*^	26.97±14.56^*^	2.162	0.034
At the end of the 24^th^ week	10.02±6.92^*^	21.04±11.33^*^	6.037	0.000

Note:

* :P<0.05 compared to before treatment.

**Fig.3:**
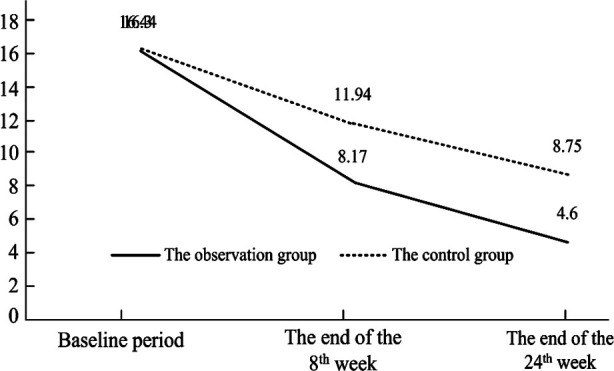
The variation trend of the SDS score of two groups in different treatment stages.

Before treatment, the SDS score did not show a significant difference between the two groups (P > 0.05). At the end of the 8th and 24th weeks after treatment, the SDS scores in both groups were significantly lower than those before treatment (P<0.05); the SDS score of the observation group was significantly lower than that of the control group (P<0.05) (Table-III and Fig.3).

**Table III:** Comparison of SDS score between two groups in different treatment stages (χ¯±Spoint).

Treatment stage	Observation group (n=90)	Control group (n=90)	t	p
Before treatment	16.30±7.73	16.43±5.86	0.108	0.916
At the end of the 8^th^ week	8.16±6.08^*^	11.93±5.94^*^	3.217	0.002
At the end of the 24^th^ week	4.61±3.48^*^	8.76±5.25^*^	4.776	0.000

Note:

* :P<0.05 compared to before treatment.

In the observation group, there were four cases of lethargy, four cases of nausea and vomiting, two cases of weight gain, and one case of abnormal liver function test, and the incidence of adverse reactions was 12.2% (11/90); in the control group, there were four cases of lethargy, two cases of nausea and vomiting, two cases of weight gain, and two cases of abnormal liver function test, and the incidence of adverse reactions was 11.1% (10/90). There was no significant difference in the incidence of adverse reactions between the two groups (x^2^=0.049, P>0.05).

## DISCUSSION

There are cognitive impairment and social function impairment in patients with depressive disorder. At present, the clinical efficacy of most antidepressants is relatively single and limited. Vortioxetine has a multi-mode mechanism of action, which can treat a wider spectrum of clinical symptoms of depression, with good safety and tolerance. At present, among limited foreign studies, the results have verified that voltaicetine can effectively improve the clinical symptoms of patients with depression.^19^ Thase et al. compared the efficacy of vortioxetine (10 mg/d) and placebo in treating 18~75 years old patients with depression who have had the disease for at least three months after six to eight weeks of treatment using the Meta analysis.^20^ The Montgomery-Asberg Depression Rating Scale (MADRS) was used as the main evaluation indicator. In USA, the effective rate of voltaicetine was 48.8% (the reduction of MADRS score ≥ 50% compared to the baseline), and the remission rate was 30.2% (MADRS ≤ 10), significantly higher than those of the placebo (36.7% and 23.8%). In the clinical trails outside USA, the efficacy of voltaicetine was 57.3%, and the remission rate was 35.7%, which were also significantly higher than those of the placebo (36.6% and 23.8%). This study found that voltaxetine and escitalopram could significantly relieve the depression and anxiety of patients with depressive disorder. The follow up at 8th and 24th weeks after treatment showed that the effects of the two drugs on the improvement of depressive symptoms in patients with depressive disorders were generally consistent. The above results indicated that both voltacetin and escitalopram could effectively treat the depression symptom of patients with depressive disorder and their curative effects were nearly the same.

For the treatment of depression, it is far from enough to control the symptoms of depression and anxiety. The cognitive impairment of most patients with depressive disorder does not decrease with the disappearance of depressive symptoms. The recovery of cognitive symptoms and social function is an important link to promote patients to return to society and adapt to the environment and obtain a good prognosis. There is an evidence that 5-HT system improves cognitive functions including learning and memory by activating or inhibiting different subtypes of 5-HT receptor.^21^ According to the fact that vortioxetine acts on 5-HT system by multi-mode, it was speculated that the drug might have the effect of improving cognitive function. Previous animal experimental studies have verified that mice treated with tryptophan hydroxylase inhibitor (methyl 4-chloro-3-phenyl-DL-alaninate hydrochloride, PCPA) lack 5-HT in vivo, and the memory damage caused can be repaired by voltahexidine but cannot be repaired by escitalopram.^22^ A randomized, controlled study of Wang et al. found that voltaxetine could significantly improve the patient’s work, social life, and home life/family responsibilities.^23^

Yet another study regarding efficacy of escitalopram in combination with hyperbaric oxygen had showed that in the treatment of depression, it results in rapid efficacy and has some effect in improving cognitive function.^24^

At present, there are only few studies in China, which only observed and studied the improvement of clinical symptoms of patients with depressive disorder by voltaxetine, and the study on the cognitive function of patients is absent. Therefore, this study focused on the improvement of cognitive function of patients with depressive disorder. The results of this study showed that both fluoxetine and escitalopram could effectively improve the social and cognitive functions of patients with depressive disorder with the advancement of treatment and the improvement of clinical symptoms. However, compared with escitalopram, voltaxetine had more obvious improvement effect on social function and cognitive function in patients with depressive disorder, which might be related to the effect of voltaxetine on multiple subtypes of 5-HT and the remodeling of hippocampal system.^25^

Some studies have pointed out that the treatment safety of voltaxetine is relatively high. For example, there is a study in the United States involving 447 patients with depressive disorder, and the adverse reactions of voltaxetine and escitalopram in sexual function were compared. The results showed that voltaxetine had less damage to sexual function and pointed out that the biggest adverse reaction of voltaxetine was nausea.^26^ In this study, there was no significant difference in the incidence of adverse reactions between fvoltaxetine and escitalopram, and the adverse reactions were mild and short-term, easy to be tolerated by patients, indicating that the treatment safety of voltaxetine was high.

## CONCLUSION

Voltaxetine can effectively and safely improve the clinical symptoms and cognitive function of patients with depressive disorder. Compared with escitalopram, voltaxetine can improve the social function and cognitive function of patients more significantly and has high safety, which is worth further clinical promotion.

### Authors’ Contribution:

**SCS & HQS:** Study design, data collection and analysis.

**BMS:** Manuscript preparation, drafting and revising.

**HQS:** Review and final approval of manuscript.

## References

[1] Liu S, Li C, Shi Z, Wang X, Zhou Y, Liu S (2017). Caregiver burden and prevalence of depression, anxiety and sleep disturbances in Alzheimer's disease caregivers in China. J Clin Nurs.

[2] Olesen J, Gustavsson A, Svensson M, Wittchen HU, Jonsson B (2012). The economic cost of brain disorders in Europe. Eur J Neurol.

[3] Smith K (2014). Mental health:a world of depression. Nature.

[4] Si TM, Fang YR, Li T, Xu XF, Hao W, Xu YF (2014). Consensus of mirtazapine in treatment of depression. Chin Mental Health J.

[5] Xiao L, Feng L, Zhu XQ, Wang G, Wu W, Hu Y (2017). A national survey of residual symptoms in Chinese depressive patients after acute phase treatment. Chin J Psychiatry.

[6] Zhang Y, Li G, Wang RM, Liu LR (2019). Cognitive impairment in patients with unipolar depression during remission. J Int Psychiat.

[7] Papakostas GI (2014). Cognitive symptoms in patients with major depressive disorder and their implications for clinical practice. J Clin Psychiatry.

[8] Shilyansky C, Williams LM, Gyurak A, Harris A, Usherwood T, Etkin A (2016). Effect of antidepressant treatment on cognitive impairments associated with depression:A randomised longitudinal study. Lancet Psychiatry.

[9] Wade A, Lemming OM, Hedegaard KB (2002). Escitalopram 10 mg/day is effective and well tolerated in a placebo-controlled study in depression in primary Care. Int Clin Psychophannacol.

[10] Rapaport MH, Bose A, Zheng H (2004). Escitalopram continuation treatment prevents relapse of depressive episodes. J Clin Psychiat.

[11] Brivio P, Corsini G, Riva MA, Calabrese F (2019). Chronic vortioxetine treatment improves the responsiveness to an acute stress acting through the ventral hippocampus in a glucocorti-coid-dependent way. Pharmacol Res.

[12] Baldwin DS, Chrones L, Florea I, Nielsen R, Nomikos GG, Palo W, Reines E (2016). The safety and tolerability of vortioxetine:Analysis of data from randomized placebo-controlled trials and open-label extension studies. Psychopharmacol.

[13] Jacobsen PL, Mahableshwarkar AR, Chen Y, Chrones L, Clayton AH (2015). Effect of vortioxetine vs escitalopram on sexual functioning in adults with well-treated major depressive disorder experiencing SSR I-induced sexual dysfunction. J Sex Med.

[14] Cipriani A, Furukawa TA, Salanti G, Chaimani A, Atkinson LZ, Ogawa Y (2018). Comparative efficacy and acceptability of 21 antidepressant drugs for the acute treatment of adults with major depressive dis-order:A systematic review and network meta-analysis. Lancet.

[15] Wang C (2019). Clinical efficacy and safety evaluation of vortioxetine hydrobromide in the treatment of major depressive disorder. Beijing Med J.

[16] Hu XQ, Qian MC, Lin M, Wang SL, Yang CH, Chen W (2017). Validity and reliability of the Chinese version of Snaith-Hamilton Pleasure Scale (SHAPS)in assessment of patients with depression. Chin Mental Health J.

[17] Liu H, Zhang HY, Xiao WD, Liu Q, Wang G, Chen JX (2015). Comparison of 5 assessment tools for evaluating depressive symptom in patients with schizophrenia. Chin Mental Health J.

[18] Fehnel SE, Forsyth BH, DiBenedetti DB, Danchenko N, François C, Brevig T (2016). Patient-centered assessment of cognitive symptoms of depression. CNS Spectr.

[19] Betry C, Pehrson A, Etievant A, Ebert B, Sánchez C, Haddjeri N (2013). The rapid recovery of 5-HT cell firing induced by the antidepressant vortioxetine involves 5-HT3 receptors antagonism. Int J Neuropsychopharmacol.

[20] Thase ME, Mahableshwarkar AR, Dragheim M, Loft H, Vieta E (2016). A meta-analysis of randomized, placebo-controlled trials of vortioxetine for the treatment of major depressive disorder in adults. Eur Neuropsychopharmacol.

[21] D'Agostino A, English CD, Rey JA (2015). Vortioxetine (Brintellix):A new serotonergic antidepressant. Pharm Therapeutics.

[22] Du Jardin KG, Jensen JB, Sanchez C, Pehrson A (2014). Vortioxetine dose dependently reverses 5-HT depletion-induced deficits in spatial working and object recognition memory:A potential role for 5-HT1A receptor ageism and 5-HT3 receptor antagonism. Eur Neuropsychopharmacol.

[23] Wang S, Wang GH, Liu XB, Jiang T, Dong XJ (2019). Clinical efficacy of vortioxetine in treatment of depressive disorder. Neur Injury Funct Reconstr.

[24] Mi K, Guo Q, Xu BY, Wang M, Bi H (2021). Efficacy of hyperbaric oxygen combined with escitalopram in depression and its effect on cognitive function. Pak J Med Sci.

[25] Lee Y, Rosenblat JD, Lee J, Carmona N, Subramaniapillai M, Shekotikhina M (2018). Efficacy of antidepressants on measures of workplace functioning in major depressive disorder:A systematic review. J Affect Disord.

[26] Jacobsen PL, Mahableshwarkar AR, Chen Y, Chrones L, Clayton AH (2015). Effect of vortioxetine vs escitalopram on sexual functioning in adults with well-treated major depressive disorder experiencing SSRI-induced sexual dysfunction. J Sex Med.

